# Neuropeptides as mediators of the early-life impact on the brain; implications for alcohol use disorders

**DOI:** 10.3389/fnmol.2012.00077

**Published:** 2012-07-05

**Authors:** Ingrid Nylander, Erika Roman

**Affiliations:** Department of Pharmaceutical Biosciences, Neuropharmacology Addiction and Behaviour, Uppsala UniversityUppsala, Sweden

**Keywords:** maternal separation, early-life environment, dynorphin, enkephalin, endogenous opioids, oxytocin, vasopressin, addiction

## Abstract

The brain is constantly exposed to external and internal input and to function in an ever-changing environment we are dependent on processes that enable the brain to adapt to new stimuli. Exposure to postnatal environmental stimuli can interfere with vital adaption processes and cause long-term changes in physiological function and behavior. Early-life alterations in brain function may result in impaired ability to adapt to new situations, in altered sensitivity to challenges later in life and thereby mediate risk or protection for psychopathology such as alcohol use disorders (AUD). In clinical research the studies of mechanisms, mediators, and causal relation between early environmental factors and vulnerability to AUD are restricted and attempts are made to find valid animal models for studies of the early-life influence on the brain. This review focuses on rodent models and the effects of adverse and naturalistic conditions on peptide networks within the brain and pituitary gland. Importantly, the consequences of alcohol addiction are not discussed but rather neurobiological alterations that can cause risk consumption and vulnerability to addiction. The article reviews earlier results and includes new data and multivariate data analysis with emphasis on endogenous opioid peptides but also oxytocin and vasopressin. These peptides are vital for developmental processes and it is hypothesized that early-life changes in peptide networks may interfere with neuronal processes and thereby contribute the individual vulnerability for AUD. The summarized results indicate a link between early-life rearing conditions, opioids, and ethanol consumption and that the ethanol-induced effects and the treatment with opioid antagonists later in life are dependent on early-life experiences. Endogenous opioids are therefore of interest to further study in the early-life impact on individual differences in vulnerability to AUD and treatment outcome.

## Introduction

The brain is constantly exposed to external and internal input and to be able to function in an ever-changing environment we are dependent on processes that enable the brain to adapt to new stimuli. The ability to adapt to new conditions is particularly important during development. The developmental processes throughout the prenatal period and the final postnatal reorganization and maturation include a variety of adaptation processes that shape brain function (Crews et al., 2007; Crews, 2008; Turrigiano, 2008). During these developmental time windows the brain is highly sensitive to environmental input. Environmental factors interact with the genome through epigenetic or transcriptional mechanisms and result in long-term alterations in basal function and neuroplasticity (de Kloet et al., 2005; Holmes et al., 2005; Crews, 2008). Such early-life alterations may be favorable for the individual but may also result in impaired ability to adapt to new situations and in altered sensitivity to challenges later in life and thereby contribute to the individual vulnerability for later disease (Nemeroff, 2004; Gluckman et al., 2007; McCrory et al., 2011).

Environmental influences, particularly during childhood and adolescence, have profound impact on the liability to develop alcohol use disorders (AUD) (De Bellis, 2002; Langeland et al., 2004). However, the mechanisms and mediators of the early-life impact on development of AUD are poorly understood. The transition from habitual drug taking into the addictive state may be affected but the mechanisms underlying the individual differences in these transition processes are not clear (Everitt and Robbins, 2005; Koob and Volkow, 2010). Studies of causal relationships between early environmental factors and later vulnerability or resilience to addiction are restricted in clinical research. How can we, for example, distinguish and establish relations between the impact of innate factors, early-life adversity, and early-life drug consumption in an individual that have been diagnosed AUD? To that end we need valid animal models where we can simulate early-life adverse and naturalistic conditions, respectively, and study the consequences later in life. The present article focuses on rodent models and environmentally induced changes in peptide networks within the brain and pituitary gland after exposure to maternal separation (MS) for short or prolonged periods during the postnatal period. Several lines of evidence from rodent MS studies support the notion that the early-life rearing conditions have long-term consequences for ethanol consumption (Weinberg, 1987; Hilakivi-Clarke et al., 1991; Huot et al., 2001; Ploj et al., 2003a; Jaworski et al., 2005; Gustafsson and Nylander, 2006). The results are not conclusive and it is evident that the effects on ethanol consumption are highly dependent on the experimental paradigm (Jaworski et al., 2005; Roman and Nylander, 2005; Moffett et al., 2007). However, most studies report higher voluntary consumption after prolonged separations as compared to the low ethanol consumption seen after short periods of MS (see reviews by Roman and Nylander, 2005; Moffett et al., 2007). The results summarized herein show that environmentally induced changes in opioid networks can contribute to differences in ethanol consumption patterns later in life and that the variability in outcome may relate to the fact that different MS paradigms cause distinct effects on basal and ethanol-induced effects on opioid peptides.

Importantly, we do not focus on the addicted brain and the alterations discussed are not consequences of compulsive ethanol use. The emphasis is on the neurobiological alterations induced by early-life conditions as a cause for risk consumption and vulnerability to AUD. The article provides a review of earlier results and also includes new data from experimental studies that investigated the impact of early-life conditions on neuropeptides. Emphasis is on endogenous opioid peptides and to some extent oxytocin and vasopressin. These peptides are known to be vital for normal social, emotional, and neurobiological development (Nelson and Panksepp, 1998) and this review summarizes findings showing that early-life experiences induce pronounced changes in basal levels of these peptides, especially the opioid peptide Met-enkephalinArg^6^Phe^7^ (MEAP) and oxytocin. Furthermore, it is shown that the ethanol-induced effects on opioids and the efficacy of opioid antagonists to reduce voluntary ethanol consumption are dependent on early-life history. The compiled results support the hypothesis that early-life changes in basal opioid peptide functioning may contribute to the individual vulnerability or resilience to develop AUD.

## Targets within neuropeptide circuits

Environmental factors interact with innate factors and result in a specific response depending on the individual genotype. So far, the molecular (epigenetic and transcriptional) mechanisms underlying these interactions are unclear and, likewise, the mediators (targeted proteins) of the environmental influences are not fully known. There are a number of different proteins associated with peptide transmission and they are all putative targets for environmental influence. Peptides are synthesized in the neuron from precursor proteins and the peptides act on specific receptor proteins. A number of enzymes participate in peptide turnover; processing enzymes cleave the precursor proteins into active peptides, converting enzymes participate in the conversion of one bioactive peptide into another peptide that may have other effects through actions on another receptor, and inactivating enzymes metabolize peptides into inactive peptide fragments (Hallberg and Nyberg, 2003; Hallberg et al., 2005; Nyberg and Hallberg, 2007). Environmental influences may cause altered activity in any of the genes encoding for the precursor proteins, the receptors, and/or the enzymes. Changes in any of these proteins may contribute to altered activity in neuronal circuits that utilize peptides as transmitters and in networks that are modulated by peptides. The present review focuses on the effects on the products of the genes with emphasis on peptides, particularly the opioid peptides.

### Endogenous opioids

After the first reports of endogenous opioid receptors (Pert and Snyder, 1973; Simon et al., 1973; Terenius, 1973) three distinct receptors have been described, the mu-opioid peptide receptor (MOPR), the delta-opioid peptide receptor (DOPR), and the kappa-opioid peptide receptor (KOPR) (Kieffer and Evans, 2009). Opioid receptors are widely distributed throughout the neuroaxis, and although the localization is similar to some extent, the different receptor types display specific anatomical distributions (Mansour et al., 1988; Akil et al., 1998; Kieffer and Evans, 2009). The opioid peptide family includes endorphins, enkephalins (ENKs), and dynorphins (DYNs) and they bind with different selectivity to MOPR, DOPR, and KOPR, respectively (Akil et al., 1998; Terenius, 2000). Endogenous opioids originate from the precursor proteins proopiomelanocortin (POMC), proENK, and proDYN (Nakanishi et al., 1979; Kakidani et al., 1982; Noda et al., 1982). Each precursor protein is the product of a distinct gene and is enzymatically cleaved to several peptide products. ProENK is the precursor of multiple ENKs such as Leu-ENK, Met-ENK, and MEAP. ProDYN generates dynorphin A (DYNA), dynorphin B (DYNB), and neoendorphin. The main sites for POMC biosynthesis are the pituitary gland that releases opioids into the circulation, and the hypothalamus in neurons that project to a number of other brain areas (Smith, 2006). Neurons expressing proENK and proDYN, respectively, form pathways that are found on almost all levels in the central nervous system (CNS) and in the pituitary gland (McLaughlin, 2006; Spampinato, 2006). Co-expression of neuropeptides is common; DYNs are for example co-expressed with vasopressin in the neurosecretory hypothalamic neurons that project to the neurointermediate lobe of the pituitary gland (Summy-Long et al., 1984). The opioids are involved in basal functions such as motivation, reproductive behavior, food and fluid intake, but also in analgesia, stress reactivity, learning and memory, reward and reinforcement, motor function, and endocrine regulation (Van Ree et al., 1999; Kieffer and Gaveriaux-Ruff, 2002; Przewlocki, 2002; Trigo et al., 2010; Bodnar, 2011). The opioid peptides also serve as neuromodulators and the opioid regulation of dopaminergic pathways (Christensson-Nylander et al., 1986; Spanagel et al., 1992; Steiner and Gerfen, 1998) is of special interest with respect to drug reward, reinforcement, and addiction. The opioid receptors mediate different, often opposite, effects on dopamine transmission. Activation of the KOPR is for example associated with dysphoria whereas the MOPR and DOPR mediate euphoric effects (Akil et al., 1998) and KOPR activation results in reduction in extracellular levels of dopamine in the nucleus accumbens whereas MOPR agonists increase dopamine, presumably through actions in the VTA (Shippenberg et al., 1992; Spanagel et al., 1992; Herz, 1998; Zapata and Shippenberg, 2006). DYNs also regulate the striatonigral dopamine pathway and here DYNB has been suggested to exert negative feedback control, a specific effect that is distinct from that of the ENKs (Christensson-Nylander et al., 1986; Herrera-Marschitz et al., 1986). The enzymatic conversion of DYNs to Leu-enkephalinArg^6^ (DYN1-6) and Leu-ENK is therefore of special interest since it results in loss of KOPR-mediated effects in favor of DOPR-mediated effects (Hallberg et al., 2005). Consequently, any change in this enzymatic step will also change the physiological output from proDYN circuits.

The endogenous opioid peptides are present in the CNS well before birth and are among the first neurochemical markers to be detectable in the developing brain (Tohyama, 1992). During the first few weeks of neonatal life a significant developmental reorganization of the opioid systems occurs accompanied by regional divergence within the brain (McDowell and Kitchen, 1987; Morita, 1992; Leslie and Loughlin, 1993). The MOPR and KOPR are the first binding sites to appear within the rodent CNS at the embryonic stage. During the first few weeks of life the number of MOPR and KOPR increases substantially before declining to adult levels whereas the DOPR are expressed later with the highest expression during the second postnatal week (Spain et al., 1985; Petrillo et al., 1987). The presence of opioids when neurogenesis, neuronal migration, process outgrowth, and synaptogenesis occur suggests a role for opioids in neuronal developmental processes.

Opioids modulate social behavior, especially social interactions early in life, and several lines of evidence support a functional role of opioids in parental behavior and social bonding processes (Panksepp et al., 1980, 1994; Nelson and Panksepp, 1998). The finding of deficient attachment behavior in mice lacking the MOPR further strengthened the concept of opioid involvement in infant attachment behavior (Moles et al., 2004). Mouse pups lacking the MOPR gene had altered attachment behavior toward their mothers. These knockout mouse pups emitted less ultrasonic vocalizations when removed from the mother and, in addition, the preference toward their mothers' cues was abolished (Moles et al., 2004).

### Oxytocin and vasopressin

The oxytocin and vasopressin receptors are detected in the hypothalamus and the amygdala but also in other brain areas (Barberis and Tribollet, 1996; Gimpl and Fahrenholz, 2001). So far, one oxytocin receptor and three vasopressin receptors have been described (Jard et al., 1987; Kimura et al., 1992; Lolait et al., 1995; Morris, 2006). The genes encoding oxytocin and vasopressin are present on the same chromosome and the precursor proteins are predominantly expressed in neurons within the paraventricular and supraoptic nuclei of the hypothalamus with projections to a number of target areas (Buijs, 1983; Gimpl and Fahrenholz, 2001; Landgraf and Neumann, 2004). However, expression also occurs in the amygdala and other brain areas (De Vries and Buijs, 1983; Planas et al., 1995; Chodobski et al., 1998; Hallbeck et al., 1999; Morris, 2006). The magnocellular neurons project to the neurointermediate lobe of the pituitary gland and release peptides into the circulation and the parvocellular neurons project to the median eminence and release peptides into the portal circulation (Buijs, 1992; Gimpl and Fahrenholz, 2001). Neuropeptides may also diffuse over long distances in the extracellular fluid (Landgraf and Neumann, 2004) and may interact with distant receptors.

Oxytocin and vasopressin are involved in a number of physiological and behavioral functions through peripheral and central actions (Gimpl and Fahrenholz, 2001). Central actions include involvement in memory processes (Dantzer et al., 1987), anxiety (McCarthy et al., 1996; Bale et al., 2001; Ring et al., 2006), and regulation of emotional and social behavior in males and females (Young and Wang, 2004; Heinrichs and Domes, 2008; Neumann, 2008; Skuse and Gallagher, 2009). A number of studies have investigated the role of oxytocin in prairie voles that are highly affiliative, forms enduring social bonds between mates and displays bi-parental behavior, which is contrasting to rats and mice but more similar to humans. These studies have identified a neural circuitry model of social bonding including oxytocin, vasopressin, and dopamine as important target systems (Ahern and Young, 2009; McGraw and Young, 2010). Oxytocin and vasopressin also regulate stress responses (Neumann, 2002; Kramer et al., 2003; Engelmann et al., 2004; Landgraf and Neumann, 2004) and are affected by stressful stimuli (Hashiguchi et al., 1997; Ebner et al., 2005; Aguilera et al., 2008).

Oxytocin and vasopressin networks continue to develop during the early postnatal and adolescent period (Shapiro and Insel, 1989; Buijs, 1992; Lipari et al., 2001). These peptides have important roles early in life, for example they are vital for the establishment of early social behavior and several reports describe the role for oxytocin and vasopressin in maternal behavior, mother-pup interactions, and social bonding (Insel and Shapiro, 1992a,b; Nelson and Panksepp, 1998; Insel, 2003; Carter et al., 2008; Ahern and Young, 2009; McGraw and Young, 2010). Early-life experiences may interfere with these developmental processes and result in long-term neurobiological and behavioral consequences. Altered levels have for example been detected in children that have experienced early adversity (Fries et al., 2005; Heim et al., 2009).

## Animal models for studies of early-life impact on biology and behavior

The environmental conditions during the first postnatal weeks are critical for a normal development in the rodent. The first two postnatal weeks, starting within a few days after birth, are referred to as the stress hyporesponsive period since the adrenal responses to stress are blunted with little or no corticosterone release and the glucocorticoid receptors undergo critical developmental stages (Sapolsky and Meaney, 1986). The newborn rat is dependent on the mother for survival and normal development and the level of maternal licking and grooming behavior toward the pups during the first postnatal week has profound impact on their adult hypothalamus-pituitary-adrenal (HPA) axis response to stress (Levine, 2002; Weaver et al., 2004; Holmes et al., 2005). Other developmental processes are also affected; 360 min daily MS alter for example the ultrasonic vocalization trajectory (Ploj et al., 2003b) and delay eye-opening (Ploj et al., 2002). The interaction between the CNS and the endocrine system is manifested during development and may serve to “program” behavioral and neuroendocrine functions (de Kloet et al., 2005). Disturbance of the social interactions during these first postnatal weeks may therefore interfere with critical developmental processes and result in persistent changes in brain function and behavior and thereby determining the adult phenotype.

### Early handling/neonatal handling and maternal separation

Early studies showed positive effects in adult rats that had been handled daily for 10 min during the first three weeks of life (Weininger, 1954) and that daily separations of mother and pups for only three minutes, “early handling,” reduced physiological responses to stress (Levine, 1957; Levine and Lewis, 1959). Several later studies confirmed that rats subjected to brief handling displayed a hyporesponsive stress response as compared to non-handled rats (Ader and Grota, 1969; Anisman et al., 1998; Meaney, 2001; Pryce and Feldon, 2003). These studies initiated extensive research on the effects of early-life events in rodents and they were also the starting point for the use of MS as an experimental model in studies on the biological and behavioral consequences of early environmental factors.

**Figure 1:**
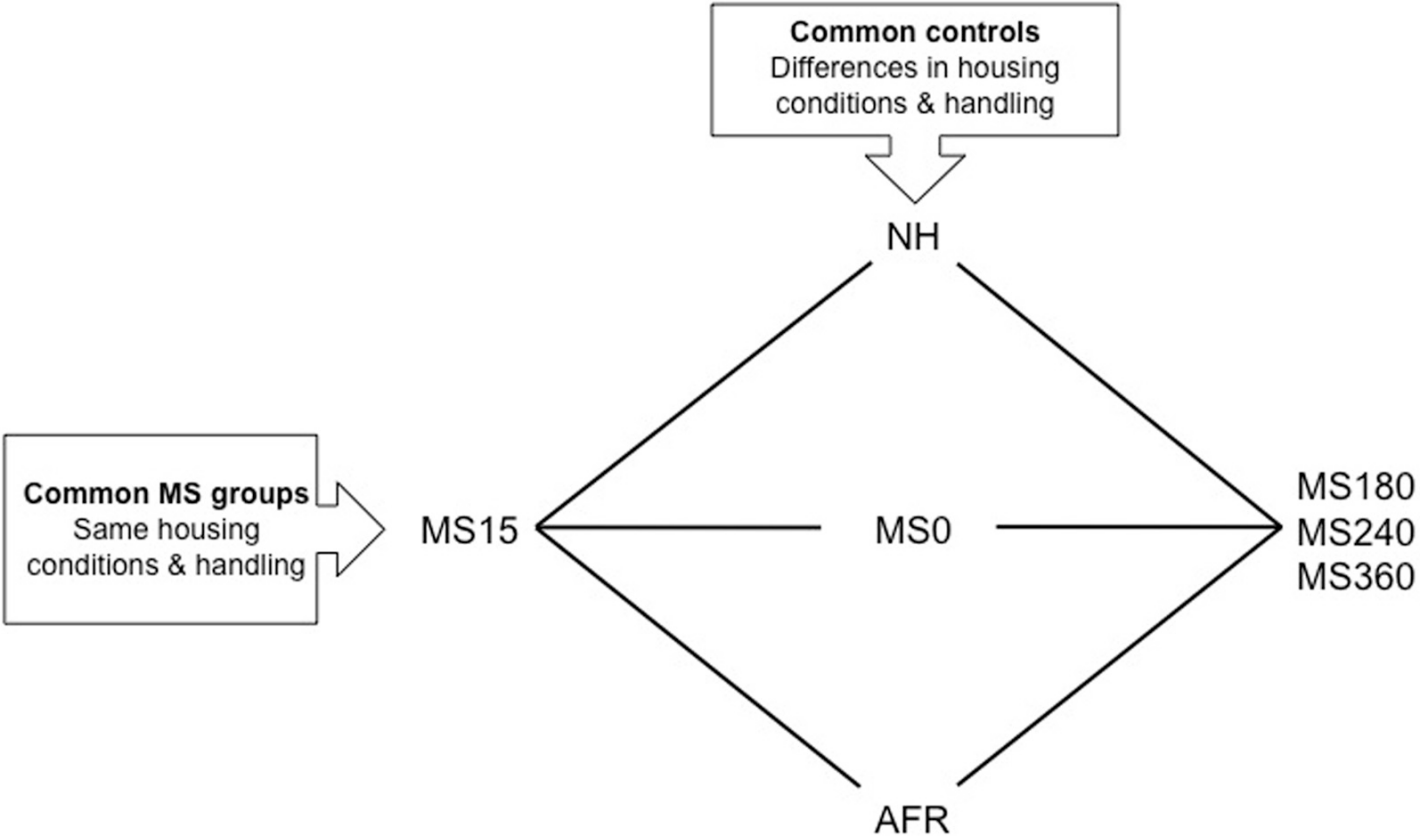
**A schematic outline of different experimental groups in rodent maternal separation (MS) paradigms**. Rats in the horizontal groups are subjected to the same handling by the experimenter and the same housing conditions. With the use of these groups the effect of being separated from the dam different length of time can be assessed. The rat pups can be exposed to either absence of the dam (litter-wise MS) or absence of the dam and the littermates (individual MS). The vertical groups represent examples of control groups commonly used in MS studies. These groups differ in a number of aspects and care must be taken in choosing the proper control depending on the question asked. NH, non-handling; AFR, animal facility rearing; MS0, brief handling of the pup; MS15, 180, 240, 360, maternal separation for 15, 180, 240, or 360 min.

In MS models, the separation of the mother and the pups is commonly performed during the first two to three postnatal weeks. Both shorter and prolonged separations are denominated MS in the literature, for example MS15 for a short 15 min separation and MS180, MS240, or MS360 for 180–360 min separations (Figure 1). Maternal deprivation usually refers to 24 h of maternal absence. The experimental design in MS paradigms aims to create early adversity by repeatedly separating the rat pups from the dam for longer periods of time, commonly 180 min or longer. Prolonged separations disrupt the early social mother-pup interactions that are vital for normal neuronal and behavioral development and are regarded as a risk environment associated with early-life stress and negative consequences later in life (Ladd et al., 2000; Levine, 2002; Pryce and Feldon, 2003; Holmes et al., 2005).

Although a number of MS protocols are in use and several authors have described the problem with the variability in outcome (Lehmann and Feldon, 2000; Pryce and Feldon, 2003; Jaworski et al., 2005; Roman and Nylander, 2005) the accumulated knowledge from these studies has improved our general understanding of the effects of various early-life conditions on the brain and behavior. The use of different control groups when assessing the consequences of prolonged MSs is one reason for variable results (see e.g., Lehmann and Feldon, 2000; Levine, 2002; Pryce and Feldon, 2003; Roman and Nylander, 2005; Macri and Würbel, 2006). Common control groups and MS groups are depicted in Figure 1. Non-handling refers to a condition with no experimenter contact during the first postnatal weeks and no cleaning/changes of the cages. Animal facility rearing (AFR) refers to conventional animal facility housing with experimenter contact only when the cages are changed. Although conventional housing is similar between laboratories there are still several possible confounding factors due to different laboratory environments. Furthermore, often it is not reported whether there are one or several experimenters participating in the separation procedures and not how the contacts are made. Non-handling and AFR are clearly not similar to a normal rodent rearing environment and the non-handling paradigm has even been suggested to be stressful (Pryce and Feldon, 2003; Macri and Würbel, 2006). The mother and pups are constantly together and the animals are subjected to few environmental stimuli compared to wildlife conditions. In addition, the handling during AFR and non-handling differs from the MS condition and it is not possible to distinguish between effects induced by handling and separation (Figure 1).

The use of briefly handled rats as control to prolonged separation reduces the problem with different handling. In the literature, brief handling procedures include MS0 that usually refers to separation less than a minute, or handling for 1–5 min (Lehmann and Feldon, 2000; Pryce and Feldon, 2003; Jaworski et al., 2005; Roman and Nylander, 2005). Based on the similarity to wildlife rearing conditions where the lactating dam leaves the nest regularly, often around 10 min and not longer than one hour depending on the age of the offspring (Grota and Ader, 1969), repeated shorter separations (3–15 min) are commonly used to simulate a beneficial environment related to positive behavioral consequences in studies of protective factors. Comparisons between short and prolonged separations also enable analysis of the effects of the duration of maternal absence without confounding effects of other experimental conditions, such as handling by the experimenter and general housing conditions (Figure 1).

One example of different outcome in MS studies is the effects on the HPA axis. HPA axis function is clearly affected by early handling and separation procedures (Anisman et al., 1998; Lehmann and Feldon, 2000; Pryce et al., 2002, 2005; Macri and Würbel, 2006) but the effects are not conclusive and highly dependent on the control group (see Pryce and Feldon, 2003; Macri and Würbel, 2006). Early handling results in a hyporesponsive HPA axis response compared to non-handling whereas prolonged separations results in a hyperresponsive HPA axis but only in comparison to early handling and not compared to non-handling (Ladd et al., 2000; Lehmann and Feldon, 2000; Meaney, 2001; Pryce and Feldon, 2003; Nemeroff, 2004). More recent studies have shown a blunted corticosterone response after prolonged separations compared to animals subjected to AFR or short separations (Greisen et al., 2005; Kim et al., 2005; Roman et al., 2006).

Finally, the outcome of MS is also dependent on experimental parameters such as the origin of animals. Recent studies have described profound differences in behavior (Palm et al., 2011) and in basal and ethanol-induced levels of opioids (Palm et al., 2012) in Wistar rats from different suppliers. Temperature, size, and sex composition of the litters, if separations are performed daily or at randomly chosen days, etc., are other examples of parameters that affect the outcome (Lehmann and Feldon, 2000; Pryce and Feldon, 2003; Roman and Nylander, 2005). Prolonged separations may alter maternal care and several authors have discussed whether the effects seen after MS are due to the loss of maternal contact, caused by an altered behavior directed toward the pup, or both (Liu et al., 1997; Caldji et al., 1998; Francis et al., 1999; Pryce et al., 2001; Marmendal et al., 2004; Macri and Würbel, 2006; Eklund et al., 2009; Daoura et al., 2010).

## The effects of early-life conditions on basal levels of neuropeptides

Since the effects induced by rearing in the risk environment (repeated prolonged separations) depend on whether AFR, non-handling or short repeated separations are used as controls the following section is divided into two parts, one section (“Maternal Separation vs. Non-handled Rats or AFR Rats”) describing the effects of MS in comparison to either non-handled rats or rats subjected to AFR, and the following section (“Short vs. Prolonged Maternal Separation”) showing the effects of prolonged MS compared to short MS.

### Maternal separation vs. non-handled rats or AFR rats

**Figure 2:**
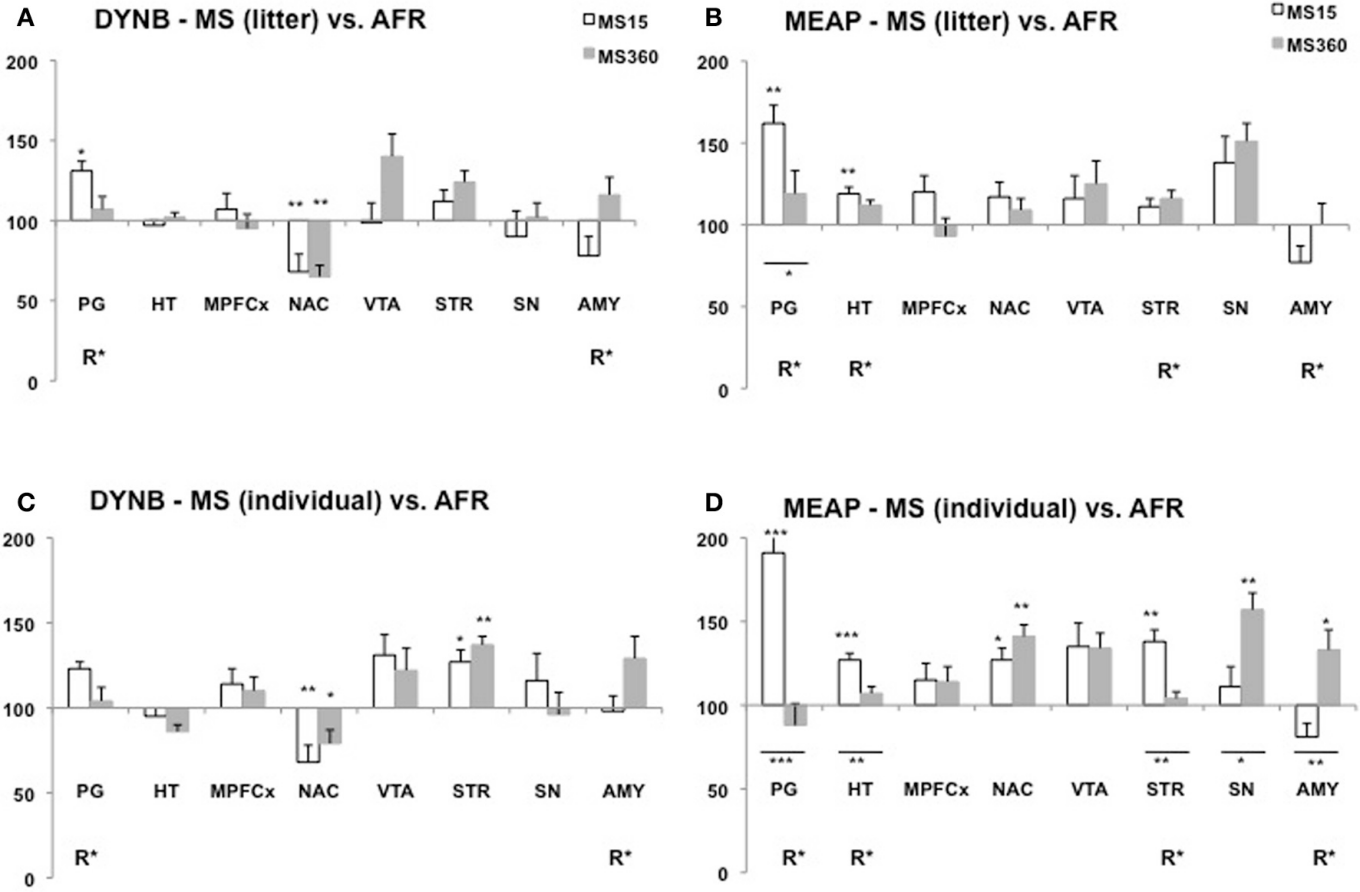
**Dynorphin B (DYNB) and Met-enkephalin-Arg^6^Phe^7^ (MEAP) in 3-week-old rats after exposure to different postnatal conditions**. Immunoreactive levels of DYNB **(A,C)** and MEAP **(B,D)** in rats subjected to MS15 or MS360 in the litter-wise MS paradigm **(A,B)** and in the individual MS paradigm **(C,D)**. The peptides were analysed at three weeks of age, immediately after the MS period. Values represent mean ± standard error of the mean and are shown as percent of the peptide levels in animals subjected to animal facility rearing (AFR) conditions (data from Gustafsson et al., 2008). ^*^p < 0.05, ^**^p < 0.01, ^***^p < 0.001. ^*^Comparison with peptide levels in the AFR group. ^*^Under a line, comparison between the MS15 and MS360 groups. R^*^, main effect of rearing environment, i.e., difference between all MS15 rats (litter-wise and individual separation) and all MS360 rats (litter-wise and individual separation). Abbreviations: PG, pituitary gland; HT, hypothalamus; MPFCX, medial prefrontal cortex; NAC, nucleus accumbens; VTA, ventral tegmental area; STR, dorsal striatum; SN, substantia nigra; AMY, amygdala.

**Figure 3:**
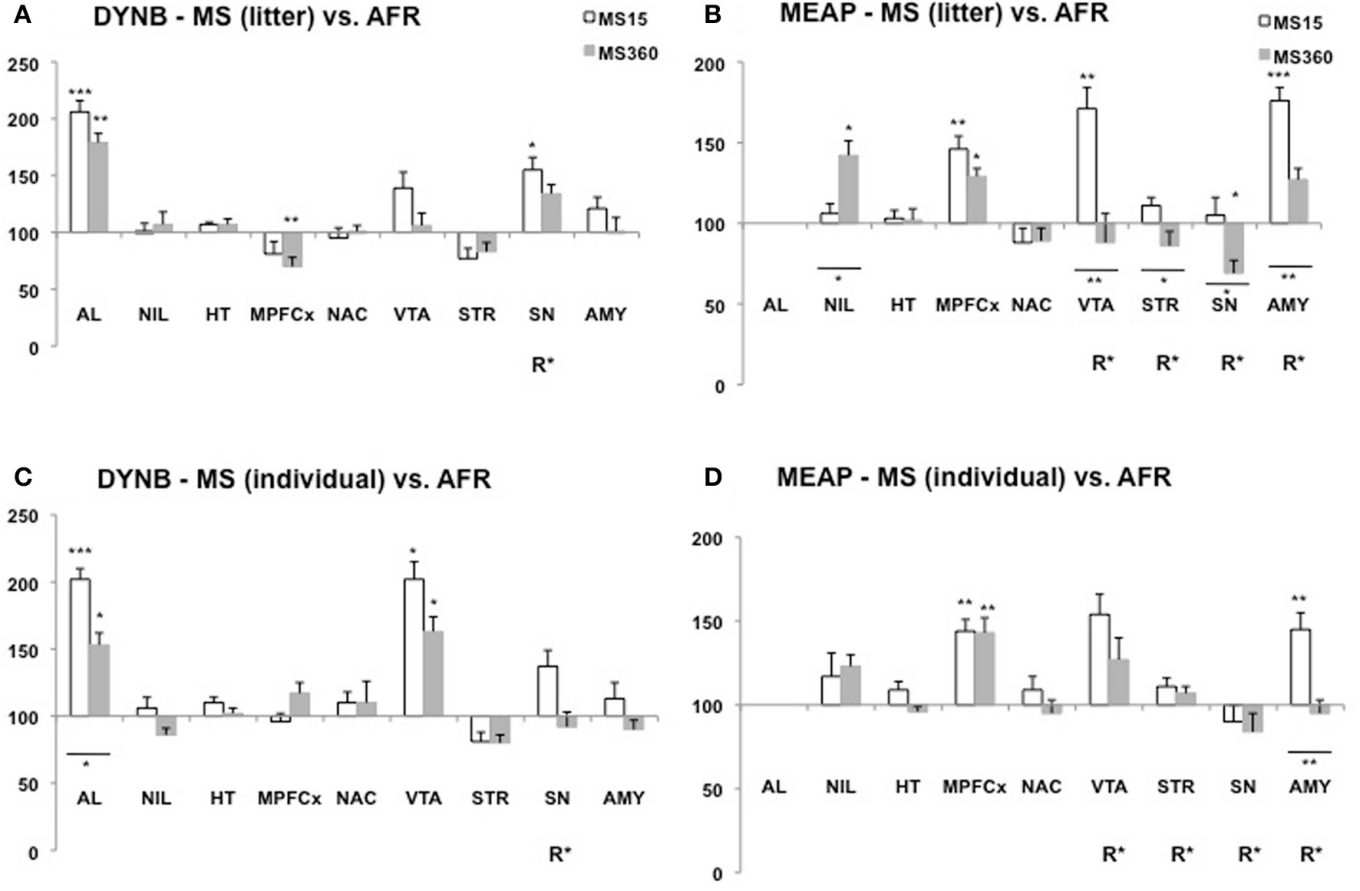
**Dynorphin B (DYNB) and Met-enkephalin-Arg^6^Phe^7^ (MEAP) in 10-week-old rats after exposure to different postnatal conditions**. Immunoreactive (ir) levels of DYNB **(A,C)** and MEAP **(B,D)** in rats subjected to MS15 or MS360 in the litter-wise MS paradigm **(A,B)** and in the individual MS paradigm **(C,D)** were analysed at 10 weeks of age, seven weeks after the MS period. Values represent mean ± standard error of the mean and are shown as percent of the levels in animals subjected to animal facility rearing (AFR) conditions (data from Gustafsson et al., 2008). ^*^p < 0.05, ^**^p < 0.01, ^***^p < 0.001. ^*^Comparison with peptide levels in the AFR group. ^*^Under a line, comparison between the MS15 and MS360 groups. R^*^, main effect of rearing environment, i.e., difference between all MS15 rats (litter-wise and individual separation) and all MS360 rats (litter-wise and individual separation). Abbreviations: AL, anterior lobe of the pituitary gland; NIL, neurointermediate lobe of the pituitary gland; HT, hypothalamus; MPFCX, medial prefrontal cortex; NAC, nucleus accumbens; VTA, ventral tegmental area; STR, dorsal striatum; SN, substantia nigra; AMY, amygdala.

Several studies show that MS affects DYNB levels and differences have been described both in comparison with non-handled rats (Ploj et al., 1999) and AFR rats (Ploj et al., 2003b; Gustafsson et al., 2008). Robust effects have been described in the pituitary gland with higher DYNB levels in adult rats after daily individual 15 min MS compared to non-handled rats (Ploj et al., 1999) and after either litter-wise or individual separations compared to AFR (Ploj et al., 2003b; Gustafsson et al., 2008). Previously described MS-induced effects on DYNB in various brain areas are not entirely conclusive (Ploj et al., 1999, 2001, 2003b) and the impact of age and different rearing conditions on the outcome was specifically addressed in a recent comparative study. The basal levels were assessed after either individual or litter-wise MS for either 15 or 360 min in 3- and 10-week-old rats in one experiment with one experimenter, the same animal housing conditions and the same rat strain and supplier (Gustafsson et al., 2008). This study revealed both immediate (Figure 2) and long-lasting (Figure 3) effects on DYNB and also distinct effects of litter-wise MS (Figures 2A,3A) and individual (Figures 2C,3C) MS, respectively, but when compared to AFR the effects were similar after MS15 and MS360. MS was again found to increase DYNB levels in the pituitary gland. In the hypothalamus no differences were detected between the MS and AFR rats. These results contrast the finding of higher DYNB after individual MS15 compared to non-handled rats (Ploj et al., 1999) and in litter-wise MS15 compared to AFR in adult single-housed rats (Ploj et al., 2003b). Interestingly this effect was clearly sex dependent; in female rats MS15 instead resulted in decreased hypothalamic DYNB (Ploj et al., 2001). These results indicate that the effects on DYNB in the hypothalamus are only detectable when an additional stress-component was added to the repeated maternal absence, such as single housing. Compared to AFR, MS induced opposite effects in the nucleus accumbens and dorsal striatum in young rats (Figure 2); a pronounced reduction in DYNB levels was seen in the nucleus accumbens both after litter-wise and individual MS whereas in the striatum, individual MS caused markedly higher DYNB levels (Gustafsson et al., 2008). In adult rats (Figure 3), differences between MS and AFR were instead found in the substantia nigra after litter-wise MS and in the ventral tegmental area (VTA) after individual MS. The different effects in the ventral and dorsal striatum and in the substantia nigra and VTA where DYNB is known to modulate the mesolimbic and nigrostriatal dopamine pathways (Christensson-Nylander et al., 1986; Herrera-Marschitz et al., 1986) indicate that handling procedures during MS may affect the opioid regulation of dopamine transmission.

The opioid peptide MEAP seems to be highly sensitive to handling and separation procedures during the postnatal period (Ploj et al., 2003b; Gustafsson et al., 2007, 2008). The effects are region-specific, dependent on separation conditions and the age when the effects were examined (Gustafsson et al., 2008). In the pituitary gland both litter-wise and individual MS15 resulted in higher MEAP levels as compared to AFR in 3-week old rats (Figure 2). In the brain, the individual and litter-wise MS resulted in distinct effects. Individual MS produced changes that were detectable in basal levels immediately after the MS period (Figure 2D); higher levels were seen after daily MS15 in the hypothalamus, nucleus accumbens, striatum, and higher levels after daily MS360 in the frontal cortex (not shown in the figure), nucleus accumbens, substantia nigra, and amygdala compared to AFR rats. Notably, litter-wise MS produced a completely different set of results; very few effects were detected in the young rats whereas measurement of peptides in adulthood revealed differences in a number of brain areas (Figure 3B). Higher levels were detected after litter-wise MS15 in the medial prefrontal cortex, VTA, and amygdala and lower levels were found in the substantia nigra after MS360 as compared to AFR rats. These results show that the effects induced by individual MS are immediate and detected in structures related to HPA axis function whereas the litter-wise MS produce changes that are detected several weeks after the MS period in areas related to reward and addiction and with a clear distinction between short and prolonged MS (see further “Short vs. Prolonged Maternal Separation”).

MS-induced effects on oxytocin networks in rodents are dependent on protocol used and age of the analysed animals. Brief handling affected the number of neurons in the paraventricular nucleus compared to AFR (Todeschin et al., 2009). Prolonged MS for 180 min resulted in increased receptor binding in hypothalamus in 8- and 16-week-old rats but not in 5-week-old rats compared to AFR (Lukas et al., 2010). However, no differences in oxytocin levels were seen after MS360 compared to AFR in 3- or 10-week-old rats (Oreland et al., 2010). Pronounced differences in oxytocin levels were instead observed between rats exposed to MS15 and AFR rats. Both individual and litter-wise MS15 were associated with higher oxytocin levels in the hypothalamus and also in the pituitary gland (Oreland et al., 2010). The MS-induced differences in the hypothalamus and pituitary gland were attenuated in adult rats and not detectable in basal levels at 10 weeks of age (Oreland et al., 2010). The amygdala was also targeted by early environmental factors and in particularly after short separations. Oxytocin levels were lower after 15 min MS compared to AFR and low levels were also seen after litter-wise MS15 in adult rats, which indicates persistent alterations in basal oxytocin levels in MS15 as compared to AFR (Oreland et al., 2010). Oxytocin levels in rats subjected to MS360 were similar to AFR (Oreland et al., 2010) and after MS180 no differences were observed in receptor binding in the amygdala as compared to AFR (Lukas et al., 2010). These studies provide evidence for a strong influence of early-life conditions on oxytocin. The peptide levels were similar after prolonged separations and AFR conditions whereas the exposure to MS15 was clearly different from AFR. Prolonged stress has been suggested to increase oxytocin activity to protect the animal from deleterious effects of stress (Carter et al., 2008). In that respect it is interesting to note that both AFR and MS360 had higher levels as compared to MS15. Considering the role of oxytocin in sensory stimulation such as warmth and touch (Uvnäs-Moberg et al., 1993; Ågren et al., 1995) it was of interest that the individual separations with repeated loss of tactile contact had a pronounced effect on the oxytocin in the amygdala.

MS also affects vasopressin but the results are not conclusive. High vasopressin mRNA levels have been reported in the paraventricular nucleus, but not in other parts of the hypothalamus, and in the bed nucleus of the stria terminalis in 5-week-old rats subjected to 180 min separation compared to AFR (Veenema and Neumann, 2009). However, no differences in vasopressin receptor binding in hypothalamus or amygdala were seen in adult rats (Lukas et al., 2010) and no differences in basal mRNA levels or vasopressin levels (Veenema et al., 2006) compared to AFR. With another protocol no differences were observed in vasopressin mRNA levels in the paraventricular nucleus after short 3 min individual separation compared to non-handled rats (Gabriel et al., 2005). Furthermore, no differences in basal vasopressin levels were observed in the pituitary gland, hypothalamus or amygdala at 3 or 10 weeks of age in rats subjected to either MS15 or MS360 compared to AFR (Oreland et al., 2010). Taken together, these studies indicate few or no differences between MS and AFR in basal levels of vasopressin.

Taken together, neuropeptide levels in rats exposed to MS clearly differ from levels in non-handled rats and rats housed according to conventional laboratory control conditions, i.e., AFR conditions. The most important take home message from the comparisons between rats subjected to short or prolonged MS vs. AFR is that the peptide levels differed between rats exposed to the shorter MS and AFR. In other words, the peptide levels in the conventional laboratory rat were different from those detected in rats reared under conditions similar to natural conditions. The outcome of MS will thus largely depend on whether AFR or short MS is used as control group. It is also worth noting that the rats that were subjected to the putative most stressful experience, i.e., daily MS360 from both the dam and the littermates, had oxytocin levels comparative to the conventional laboratory rat. These results raise the question of how “normal” the conventional laboratory rat is.

### Short vs. prolonged maternal separation

To elucidate the impact of early-life risk factors and protective factors it is more relevant to compare the effects of repeated loss of maternal contact for extended periods, i.e., adverse conditions, with effects after shorter periods of maternal absence, i.e., beneficial conditions related to naturalistic mother-offspring interactions. This comparison is not confounded by differences in experimenter handling.

In studies where the animals were single-housed generally few differences were seen in DYNB levels between adult rats subjected to MS15 and MS360 (Ploj et al., 2003b; Gustafsson et al., 2007). Lower levels were, for example, detected in the substantia nigra and higher levels in the amygdala after MS360 (Ploj et al., 2003b) and these results were confirmed also in group-housed animals (Gustafsson et al., 2008). In the group-housed adult rats, the effects were more pronounced which again show that the housing conditions *per se* can affect peptide levels and thereby confound the outcome. A comparative study that assessed the effects of individual vs. litter-wise MS and also MS15 vs. MS360 at different ages revealed that individual and litter-wise MS resulted in similar effects on DYNB (Gustafsson et al., 2008). Differences between MS15 and MS360 (a significant effect of rearing, R, in Figures 2 and 3) was observed in the pituitary gland and the amygdala in young rats and in adult rats in the substantia nigra and the periaqueductal gray (Gustafsson et al., 2008).

Pronounced differences were seen in MEAP levels between MS15 and MS360 rats and it outcome was dependent on whether individual or litter-wise MS was used (Gustafsson et al., 2008). At 3 weeks of age, differences between MS15 and MS360 were noted in the pituitary gland, hypothalamus, striatum, and amygdala (Figure 2) and in adult rats in the VTA, striatum, substantia nigra, and amygdala (Figure 3). The effect seen shortly after the separation period was particularly evident after individual MS whereas the differences seen in adult rats were noted after litter-wise separation. These results are in agreement with findings in animals that were single-housed for two months where lower levels were seen after MS360 in the hypothalamus, medial prefrontal cortex, striatum, and also periaqueductal gray (Gustafsson et al., 2007). Taken together, generally lower MEAP levels were seen in the brain after prolonged MS in the litter-wise MS paradigm.

Early-life events may also affect enzymatic activity and thereby affect the levels of bioactive peptides. One enzymatic process that is interesting in terms of drug-induced reward, reinforcement, and addictive processes is the conversion of KOPR-acting DYNs into shorter peptides that act on the DOPR (Akil et al., 1998; Hallberg and Nyberg, 2003; Hallberg et al., 2005). An environmentally induced change in this enzymatic step will change the overall output of the proDYN system (see “Endogenous Opioids”). The impact of early-life conditions on enzymatic conversion or inactivation of peptides is poorly examined and currently addressed in our laboratory. Interestingly, we observe large differences in the rate of formation of DYN1-6 in animals previously exposed to the different early-life settings. These preliminary data indicate that early-life experiences can affect enzymatic processes and thereby change the final output from a peptide system.

Pronounced differences in oxytocin levels were observed between short and prolonged MS (Oreland et al., 2010). Oxytocin levels in the MS360 rats were lower in the pituitary gland but higher in the hypothalamus and amygdala compared to the MS15 rats at 3 weeks of age. Measurement in adult rats revealed that the difference between MS15 and MS360 was persistent in the amygdala but not in the other areas. As for vasopressin, no differences were found between MS15 and MS360 after individual or litter-wise MS, neither immediately after the MS nor in adulthood (Oreland et al., 2010).

Taken together, prolonged MS had pronounced effects on MEAP and oxytocin levels. The general finding was lower MEAP levels in adult animals that had been subjected to adverse early-life conditions. The individual and litter-wise separations clearly result in different effects on MEAP; more pronounced but short-lived immediate effects were seen after individual MS and distinct long-term effects in the litter-wise paradigm. As for oxytocin, exposure to the individual separation paradigm produced more pronounced effects and in the amygdala also more long-term changes. Less consistent effects were seen in DYNB levels but the reported difference between rats that were subjected to a proposed beneficial and stressful environment, respectively, early in life may have consequences for sensitivity to challenges later in life.

### Multivariate analysis of rearing conditions and basal peptide levels

**Figure 4:**
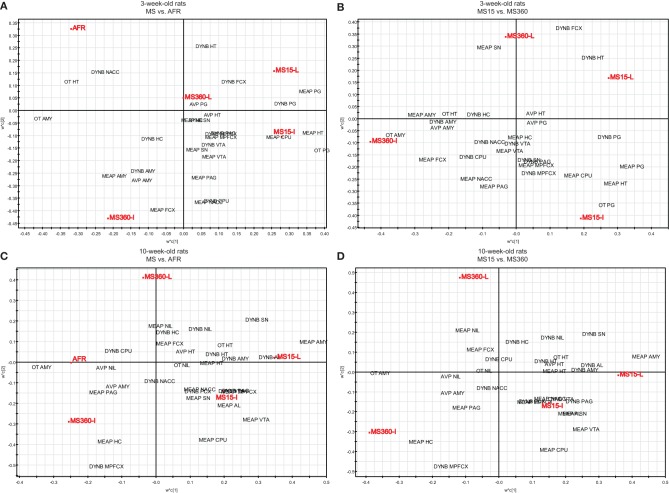
**Multivariate partial least square discriminant analysis (PLS-DA) of basal peptide levels**. PLS-DA scatter plots of basal ir peptide levels in young (**A**, R^2^X(cum) = 0.218, R^2^Y(cum) = 0.358, Q^2^(cum) = 0.233, two components and **B**, R^2^X(cum) = 0.223, R^2^Y(cum) = 0.423, Q^2^(cum) = 0.129, two components) and adult (**C**, R^2^X(cum) = 0.204, R^2^Y(cum) = 0.315, Q^2^(cum) = 0.110, two components and **D**, R^2^X(cum) = 0.201, R^2^Y(cum) = 0.457, Q^2^(cum) = 0.251, two components) rats. Rearing conditions as Y and ir peptide levels as X. Data from Gustafsson et al., 2008; Oreland et al., 2010. Abbreviations: AL, anterior lobe of the pituitary gland; AMY, amygdala; AVP, vasopressin; CPU, caudate putamen = dorsal striatum; FCX, frontal cortex; HC, hippocampus; HT, hypothalamus; MPFCX, medial prefrontal cortex; NAC, nucleus accumbens; NIL, neurointermediate lobe of the pituitary gland; OT, oxytocin; PAG, periaqueductal gray; PG, pituitary gland; SN, substantia nigra; VTA, ventral tegmental area.

To further investigate the relationship between short and prolonged MS, individual and litter-wise MS and peptide levels a multivariate data analysis was performed to analyse and illustrate the relations between all variables. The multivariate method partial least square discriminant analysis (PLS-DA) was used. PLS-DA is a regression extension of principal component analysis (PCA) and calculates the relationship between a Y-matrix (here rearing conditions) and an X-matrix (here peptide levels). The weights for the X-variables (in the analysis denoted w) indicate the importance of these variables, while the weights for the Y-variables (in the analysis denoted c) indicate which Y-variables are modeled in the respective PLS model dimensions. When these coefficients are plotted in a w × c plot, we obtain a picture showing the relationships between X and Y (Eriksson et al., 2006). This analysis included all peptide data (DYNB, MEAP, oxytocin, and vasopressin) from a large comparative study (Gustafsson et al., 2008; Oreland et al., 2010) in which the effects of daily MS15 and MS360 were assessed and compared with AFR and, in addition, examination of the influence of individual or litter-wise separations were investigated in both young and adult rats. The animals were all part of the same study with all experimental conditions exactly the same such as experimenter, laboratory housing conditions, and batch of rats (Gustafsson et al., 2008). The analysis was performed with all experimental groups including AFR (Figures 4A and C) and without AFR with only those groups exposed to the same handling included (Figures 4B and D). In the three-week old animals (Figure 4A) there is a clear separation between AFR and individually separated MS360 (in the left quadrants) and MS15 rats (in the right quadrants). Furthermore, individually separated MS15 rats load in the lower right quadrant, separated from litter-wise separated MS15 and MS360 rats. When the AFR group was excluded from the analysis (Figure 4B), all four MS conditions separate from each other, with the individually separated MS360 rats being the most extreme group. In adult animals (Figure 4C), AFR rats and MS360 rats load in the left quadrants and separate from the MS15 rats. Moreover, in the right quadrants AFR rats load in-between litter-wise and individually separated MS360 rats. Separation condition (i.e., litter-wise or individual) seems to be of less importance for adult MS15 rats. When the AFR group was excluded from the analysis (Figure 4D), a similar patter as in the young animals appear with a clear separation between litter-wise and individually separated MS360 rats, and MS15 rats. These results confirm the notion that prolonged MS is similar to the AFR condition whereas the MS15 clearly separates from both the MS360 and AFR groups. The results also show that the differences are persistent into adulthood and, finally, they confirm the notion from other analyses that short litter-wise MS, which resemble natural conditions, are clearly separated from the AFR condition.

## Implications for alcohol use disorders (AUD)

Evidence for a link between ethanol and endogenous opioids have been shown in numerous studies. Ethanol-induced opioid activation is suggested to contribute to ethanol reward and reinforcement (Nylander and Silberring, 1998; Barson et al., 2009). Opioid receptor antagonists attenuate the ethanol-induced increase in extracellular dopamine (Benjamin et al., 1993) and reduce ethanol consumption in laboratory animals (Ulm et al., 1995) and in humans (O'Malley et al., 1992; Volpicelli et al., 1995). This effect may relate to diminished ethanol-induced reward and today naltrexone is used in the treatment of AUD. Endogenous opioids have also attracted interest in terms of the etiology of AUD and for the neurobiological basis for individual differences in the propensity to develop AUD (Nylander et al., 1994; Gianoulakis, 1996; Nylander and Silberring, 1998; Modesto-Lowe and Fritz, 2005; Trigo et al., 2010). However, several possible mechanisms have been proposed for the involvement of opioids in the vulnerability/resilience for AUD (Van Ree et al., 1999; Gianoulakis, 2004; Oswald and Wand, 2004; Sanchis-Segura et al., 2005; Drews and Zimmer, 2010). One line of evidence suggests that an inherent opioid deficiency with low levels of endogenous opioids leads to enhanced ethanol consumption that stimulates opioid activity and compensate for the deficiency (Trachtenberg and Blum, 1987). Another hypothesis assumes that vulnerable individuals inherit or acquire an excess of opioid activity that leads to increased ethanol consumption (Reid et al., 1991). In addition it has been suggested that the vulnerability for increased ethanol consumption is determined by individual differences in sensitivity of the opioid system to ethanol (Gianoulakis, 1996). Here we extend the ethanol-opioid link and suggest a link between early-life environment, opioids, and ethanol consumption.

### Association between basal peptide levels and ethanol consumption after MS

In agreement with other studies (Weinberg, 1987; Hilakivi-Clarke et al., 1991; Huot et al., 2001; Ploj et al., 2003a; Jaworski et al., 2005; Gustafsson and Nylander, 2006) we have shown that adult rats previously subjected to postnatal prolonged MS have propensity for high ethanol intake and preference. Adult male MS360 rats had a higher voluntary ethanol consumption (Ploj et al., 2003a) and higher preference for 20% ethanol (Gustafsson and Nylander, 2006) in continuous access paradigms and higher ethanol intake and preference in an intermittent ethanol consumption paradigm (Daoura et al., 2011) compared to MS15 that were reared according to naturalistic conditions. These differences may relate to long-term behavioral differences induced by the respective short and prolonged repeated postnatal litter-wise separations from the dam. The adult behavioral profile of MS360 rats includes higher exploration, higher risk-assessment, and somewhat higher risk-taking behavior (Roman et al., 2006), which resembles that of ethanol-preferring AA rats (Roman et al., 2007, 2012). There are so far no indications that the high ethanol consumption in MS360 rats is a result of anxiety-related behaviors; the MS360 rats show anxiety-like behavior at three weeks of age but not in adulthood (Ploj et al., 2002; Roman et al., 2006).

Neurobiological differences between MS15 and MS360 rats may also contribute to the differences in ethanol consumption patterns. The findings so far do not support a clear association between ethanol intake and basal levels either of DYNB, oxytocin or vasopressin in animals subjected to different early-life conditions. However, as for the opioid peptide MEAP a series of studies using different MS paradigms have provided evidence for an association between the MS-induced effects on basal levels and ethanol consumption. These studies revealed that the difference in voluntary ethanol intake behavior between MS15 and MS360 was observed in one specific MS experimental set-up, i.e., the litter-wise MS paradigm and only in male rats (Ploj et al., 2003a; Gustafsson and Nylander, 2006; Daoura et al., 2011). In this set-up, adult male MS30 rats also had lower MEAP levels in brain areas related to reward, motivation, and addiction processes (Gustafsson et al., 2008). In contrast, there were no differences in the ethanol consumption (Roman et al., 2004; Gustafsson et al., 2005) and only minor neurobiological changes in these brain areas in adult female rats (Gustafsson et al., 2005). When young rats were given access to ethanol throughout adolescence there was no difference in ethanol intake between MS15 and MS360 (Daoura et al., 2011) and in young rats there were no differences in basal MEAP levels (Gustafsson et al., 2008). Finally, when the rats were subjected to individual MS instead of litter-wise MS the ethanol consumption in adult rats was similar regardless of rearing according to the MS15 or MS360 condition (Oreland et al., 2011) and only minor differences in MEAP were seen in reward-related brain areas (Gustafsson et al., 2008). Individual MS resulted in changes in areas associated with HPA axis activity in young rats but these differences between MS15 and MS360 were not persistent (Gustafsson et al., 2008) and presumably had little relevance for ethanol consumption behavior in the adult rat.

### Association between early-life conditions and ethanol-induced effects

The first responses to ethanol are important determinants for the individual drug-taking behavior (Schuckit et al., 2004). It is therefore of interest to examine whether individuals exposed to early-life risk respond differently to ethanol. An environmentally induced alteration in ethanol response during the initiation and habituation of ethanol consumption may relate to the individual liability to go from a controlled, habitual drinking to an uncontrolled and compulsive use.

It is well-established that endogenous opioids are involved in ethanol-induced actions although the exact mechanisms are not clear (Van Ree et al., 2000; Gianoulakis, 2004; Spanagel, 2009; Trigo et al., 2010). A number of studies describe the effects of ethanol on opioids but the majority of these studies examine effects after forced drinking (Schulz et al., 1980), liquid diet (Seizinger et al., 1983), injection procedures (Lindholm et al., 2000), or inhalation in a vapour chamber (Zapata and Shippenberg, 2006) and do not address the effects of long-term voluntary ethanol drinking in animals without genetic preference for ethanol. As for DYNB, a free choice between ethanol and water in adulthood resulted in enhanced levels in the anterior lobe of the pituitary gland, hypothalamus, medial prefrontal cortex, and substantia nigra whereas ethanol-induced low levels were seen in the neurointermediate lobe of the pituitary gland (Gustafsson et al., 2007). These ethanol-induced effects were similar in rats subjected to short and prolonged separations. However, a negative correlation between the intake of 20% ethanol and DYNB levels was observed in the amygdala in the MS360 rats but not MS15 rats (Gustafsson et al., 2007) which may relate to differences in ethanol intake in these rats; DYNs mediate aversive actions (Akil et al., 1998) and low DYNB levels in animals with a high ethanol intake may relate to their propensity to consume more ethanol.

**Figure 5:**
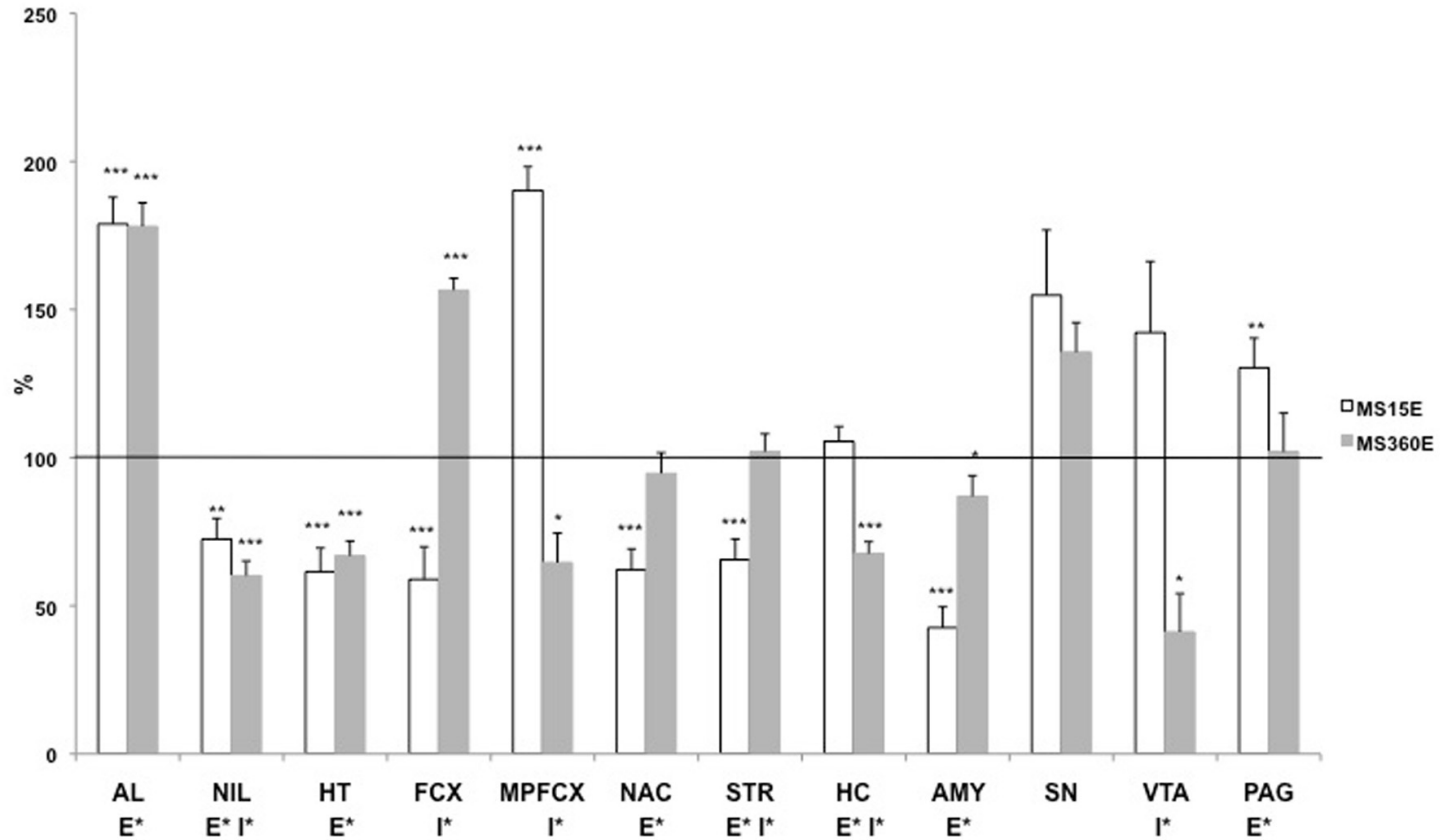
**The DYN1-6 levels in the pituitary gland and different brain areas in adult ethanol-drinking rats after exposure to different postnatal conditions**. The rats have been exposed to either MS15 or MS360 during the first three postnatal weeks and from ten weeks of age they were given free access to ethanol for eight weeks before decapitation. The levels represent immunoreactive (ir) DYN1-6 (Leu-enkephalin-Arg^6^) levels expressed as percent of the basal ir levels in water-drinking MS15 and MS360 rats, respectively. ^*^p < 0.05, ^**^p < 0.01, ^***^p < 0.001, compared to water-drinking rats. E^*^, main effect of ethanol, i.e., difference between all ethanol-drinking rats (MS15 and MS360) compared to water-drinking rats (MS15 and MS360); I^*^, interaction effect, i.e., different effect of ethanol in the MS15 and the MS360 rats (Two-Way analysis of variance). Abbreviations: AL, anterior lobe of the pituitary gland; NIL, neurointermediate lobe of the pituitary gland; HT, hypothalamus; MPFCX, medial prefrontal cortex; NAC, nucleus accumbens; VTA, ventral tegmental area; STR, dorsal striatum; SN, substantia nigra; AMY, amygdala.

Large differences were noted in the ethanol-induced effects on DYN1-6 depending on early-life environmental history (previously unpublished results; Figure 5). The DYN1-6 levels were measured in rats that were subjected to daily MS15 or MS360 and then given access to a free choice between ethanol and water for eight weeks in adulthood. The tissue levels of DYN1-6 were measured in the same animals as used for analysis of DYNB and MEAP [for detailed experimental design, see (Gustafsson et al., 2007)]. Voluntary ethanol intake affected DYN1-6 in several brain areas and in the pituitary gland (Figure 5) and the effects was more pronounced than those seen in the other opioid peptides (Gustafsson et al., 2007). An overall effect of ethanol was noted in the anterior lobe of the pituitary gland, neurointermediate lobe of the pituitary gland, hypothalamus, nucleus accumbens, striatum, hippocampus, amygdala, and periaqueductal gray. Interestingly, a statistically significant interaction between rearing condition (MS15 vs. MS360) and ethanol (ethanol vs. water) was also seen in several areas: in the neurointermediate lobe of the pituitary gland, frontal cortex, medial prefrontal cortex, striatum, hippocampus, and in the VTA (Figure 5). These results show that long-term ethanol drinking has a pronounced impact on the DYN1-6 levels and that the ethanol-induced effects in adult rats are dependent on the previous exposure to either postnatal MS15 or MS360. The effects on DYN1-6 were clearly distinct from the effects on DYNB indicating different effects on bioactive peptides derive from the same prohormone. Considering the pronounced effects described herein on DYN1-6 and the opposite effects induced by KOPR and DOPR ligands on networks involved in reward and motivation actions (Spanagel et al., 1992; Akil et al., 1998; Nylander and Silberring, 1998; Van Ree et al., 1999) further studies are warranted on different proDYN-derived peptides.

Long-term voluntary drinking of ethanol in adulthood was shown to differentially affect MEAP levels in rats previously exposed to different early-life conditions. The ethanol-induced effects were more pronounced in MS360 rats compared to the MS15 rats in several brain areas: the hypothalamus, medial prefrontal cortex, striatum, substantia nigra, and periaqueductal gray (Gustafsson et al., 2007). These results indicate that animals exposed to early adversity have an enhanced sensitivity to the effects of ethanol. Altered sensitivity to ethanol has been described in studies using ethanol-preferring rats such as the P rats (Li et al., 1998) and AA rats (Nylander et al., 1994). These results indicate similar phenotypes after selective breeding and exposure to environmental adversity. Opioids stimulate ethanol intake and it is suggested that ethanol drinking activates opioids that further trigger ethanol intake (Barson et al., 2009). A higher ethanol-induced activation of ENKs in the MS360 rats may relate to risk for an increased liability to escalate drinking.

Ethanol-induced effects in animals were examined in rats that were subjected to the same experimental conditions as in the study by Gustafsson et al. (2007). Voluntary ethanol drinking for eight weeks resulted in lower oxytocin levels and higher vasopressin levels in the neurointermediate lobe of the pituitary gland. In the hypothalamus ethanol drinking resulted in higher oxytocin levels whereas the vasopressin levels were not affected (unpublished results). However, these ethanol-induced effects on oxytocin or vasopressin were similar in rats that had been subjected to MS15 and MS360 the results provide no evidence for associations between either oxytocin or vasopressin and environmentally induced changes in ethanol consumption.

### Multivariate analysis of rearing conditions and peptide levels in water- and ethanol-drinking rats

**Figure 6:**
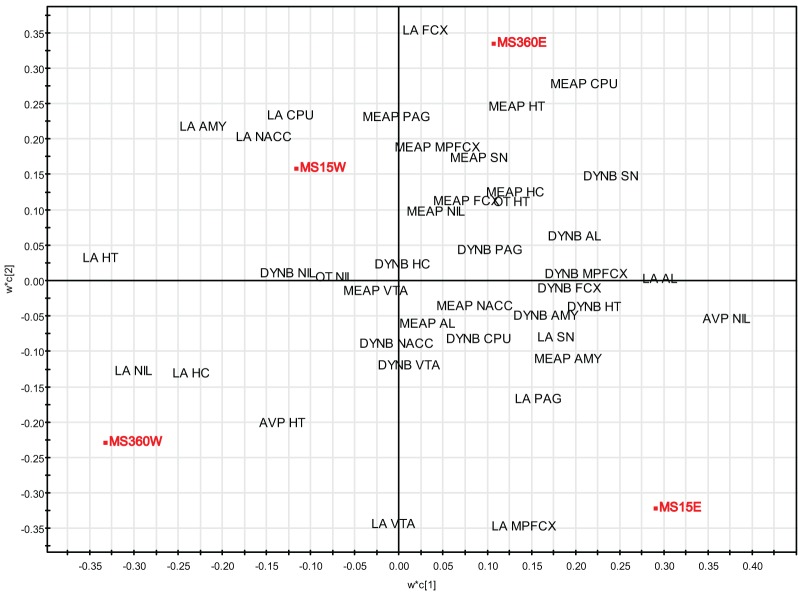
**Multivariate partial least square discriminant analysis (PLS-DA) of peptide levels in water- and ethanol-drinking rats**. PLS-DA scatter plot of the two first components (R^2^X(cum) = 0.269, R^2^Y(cum) = 0.706, Q^2^(cum) = 0.555). Rearing conditions as Y and ir peptide levels as X. Data from (Gustafsson et al., 2007). Abbreviations: AL, anterior lobe of the pituitary gland; AMY, amygdala; AVP, vasopressin; CPU, caudate putamen = dorsal striatum; FCX, frontal cortex; HC, hippocampus; HT, hypothalamus; MPFCX, medial prefrontal cortex; LA, Leu-enkephalin-Arg^6^ = DYN(1–6); NAC, nucleus accumbens; NIL, neurointermediate lobe of the pituitary gland; OT, oxytocin; PAG, periaqueductal gray; SN, substantia nigra; VTA, ventral tegmental area.

PLS-DA (for details, see section “Multivariate Analysis of Rearing Conditions and Basal Peptide Levels”) was again used in order to investigate possible relationships between rearing conditions, ethanol-induced effects, and peptide levels. Here data from a study in which opioid peptides (DYNB, MEAP, and DYN1-6), oxytocin, and vasopressin were assessed in brain areas and in the pituitary gland in rats that were given access to either water or a free choice between ethanol and water for eight weeks. The animals were all part of the same study (Gustafsson et al., 2007) with the same experimenter handling, laboratory housing conditions, and batch of rats from the supplier. The results show that the respective groups clearly load in separate quadrants with a separation between water-drinking animals in the left quadrants and ethanol-drinking rats in the right quadrants (Figure 6). Ethanol consumption in the MS360 rats changes the location from the lower to the upper quadrant whereas the opposite is seen for the MS15 groups, which indicates that different peptide profiles are affected in these experimental groups. These results confirm the notion that ethanol intake affects endogenous opioids differently depending on early-life environment, i.e., exposure to MS15 or MS360.

### Early-life conditions and the response to exogenous opioids

Early-life rearing conditions have been shown to change the effects of opioid receptor agonists and antagonists. Rats subjected to prolonged MS had an altered sensitivity to morphine (Kehoe and Blass, 1986; Kalinichev et al., 2001a,b) and enhanced naltrexone-induced suppression of sucrose consumption as compared to non-handled rats (Michaels and Holtzman, 2007). These results raised the question whether also the effects of naltrexone on ethanol consumption would be affected by early-life conditions. An increasing number of reports describe large individual variations in the effects of naltrexone in AUD patients (Kiefer et al., 2008; Spanagel and Kiefer, 2008) and pharmacogenetic studies have revealed that genetic factors contribute to individual differences in the ability of naltrexone to reduce ethanol intake (Monterosso et al., 2001; Rubio et al., 2005; Ray et al., 2010; Roman and Nylander, 2005; Moffett et al., 2007; Barr, 2011). For example, several reports have shown that MOPR polymorphism affect the response to naltrexone (Oslin et al., 2003; Mague and Blendy, 2010).

In a recent study it was confirmed that the response to naltrexone differs depending on early-life environmental conditions (Daoura and Nylander, 2011). Adult rats previously exposed to daily MS360 decreased their voluntary ethanol consumption after administration of naltrexone whereas animals subjected to MS15 did not respond (Daoura and Nylander, 2011). These results provided evidence that ethanol-drinking rats with a history of adverse early environmental experiences responded well to naltrexone, whereas rats reared in an environment related to positive behavioral consequences did not benefit from naltrexone treatment. A plausible explanation for different effects of naltrexone is the different ethanol-induced effects in MS15 and MS360 rats; MS360 rats had higher MEAP levels after voluntary ethanol consumption, whereas no ethanol-induced increase in MEAP was found in the MS15 rats (Gustafsson et al., 2007). It is therefore likely that naltrexone abolished the effect of ethanol-induced opioid activation, thereby attenuating the ethanol-induced reward and reducing ethanol intake in the MS360 rats. Similarly, the lack of ethanol-induced activation of opioids in MS15 rats may explain the poor efficacy of naltrexone in these rats. These results highlight the importance of not only considering genetic factors but also early environmental factors when identifying subtypes of AUD patients that respond well to naltrexone treatment.

Taken together, the summarized results provide evidence for a link between early-life experiences, opioids, and adult voluntary ethanol consumption. Studies so far indicate that there is no clear relation between DYNB, oxytocin or vasopressin, and ethanol consumption while there is an association between basal levels of MEAP and ethanol consumption in animals exposed to MS. In addition, differences in response to voluntary ethanol drinking and challenge with opioid agonists and antagonists after exposure to different early-life conditions have been described. Adult male rats exposed to MS360 are characterized by altered risk-taking behavior and propensity for high ethanol intake had lower central levels of MEAP and a more pronounced ethanol-induced increase in MEAP levels and they also responded with decreased ethanol consumption after naltrexone. Opioid deficiency and an enhanced sensitivity to the effects of ethanol on opioids have been proposed to be part of the neurobiological basis for increased vulnerability to addiction. Persistent changes in central ENK networks, caused by early-life experiences, may therefore contribute to the liability to go from the controlled drinking to excessive drinking and the compulsive use seen in AUD.

## Summary and future directions

The effects described herein are summarized from studies that employed rodent MS procedures to simulate different early-life settings. There are also other early-life settings, for example enrichment paradigms that are known to affect later drug consumption patterns, such as amphetamine self-administration (Bardo et al., 2001). It has also been shown that the rewarding effects of cocaine are inversely related to the degree of enrichment (Zakharova et al., 2009). Enrichment paradigms are not accounted for here and to our knowledge less is known about the effects of enrichment on peptide networks. In the present article it is shown that rearing in different early-life environments have pronounced impact on basal peptide levels, in enzymatic activity and in drug-induced effects on neuropeptides. Some effects on the peptides were immediate and seen already in the young animals and were either short lasting and attenuated, “normalized,” during adolescence or persisted into adulthood. However, the altered levels early in life point to affected peptide circuits and even though no differences could be detected in basal levels in adulthood the system may still be more sensitive to challenges later in life. Other effects appeared later in adulthood, which indicate that early environmental factors can affect neuronal networks or processes involved in peptide ontogenesis, change the developmental pattern and thereby not be detectable in basal levels until adulthood.

The summarized results show that the most pronounced effects of early-life exposure were on oxytocin and MEAP. This observation is not surprising considering that these peptide systems continue to develop and mature after birth (McDowell and Kitchen, 1987; Buijs, 1992; Lipari et al., 2001). DYN (Leslie and Loughlin, 1993) and vasopressin (Lipari et al., 2001) also continue to develop after birth but they are more fully developed at birth as compared to MEAP and oxytocin. These peptides may therefore be less sensitive to postnatal manipulations and fewer changes in basal levels of DYNB and vasopressin were also noted. However, as already mentioned, the effects of exposure to a risk environment may not be seen in basal levels but appear when the individual encounters challenge later in life such as stress, trauma, or risk consumption of drugs of abuse. DYN, for example, have been suggested to be involved in anti-reward systems in the brain (Bruijnzeel, 2009; Wee and Koob, 2010) and environmentally induced changes in DYN networks may come into play after long-term drug consumption in the addictive and withdrawal states.

Considering the vast number of physiological functions that have been described for the endogenous opioids (e.g., Van Ree et al., 1999; Kieffer and Gaveriaux-Ruff, 2002; Przewlocki, 2002; Trigo et al., 2010; Bodnar, 2011) it is evident that long-term changes in opioid functioning induced by early-life experiences will have extensive consequences for the individual. The results presented here provide evidence for a link between the early-life environment, endogenous opioids, and adult ethanol consumption. Exposure to an early-life setting associated with disturbed interactions between the caregiver and the offspring result in long-term changes in basal levels of the ENK peptide MEAP and altered response to ethanol in adult rats. Dysfunctional opioid networks and altered ethanol-induced response in rats exposed to early-life stress may relate to their high ethanol consumption and also to the high efficacy of naltrexone in reducing ethanol intake. Endogenous opioids are therefore relevant to further study as putative mediators of environmentally induced vulnerability or resilience to AUD.

Altered oxytocin levels were seen in young rats in the hypothalamus, pituitary gland and the amygdala and the repeated loss of tactile contact during the separations in the individual MS paradigm resulted in persistent effects in the amygdala. These results show that oxytocin is a target for early-life influence and show the importance of early-life tactile contact for normal oxytocin development. The differences between basal oxytocin levels in rats subjected to a risk and a preventive environment, respectively, were not associated with differences in ethanol consumption (Oreland et al., 2010) and the ethanol-induced effects were similar.

However, considering the reports of links between oxytocin, early-life behavior and mental health (Panksepp, 1992; Meyer-Lindenberg, 2008) it is likely that environmentally induced changes in oxytocin functioning will affect later behavior. Oxytocin has anxiolytic effects (McCarthy et al., 1996; Bale et al., 2001; Ring et al., 2006), is involved in stress-coping behavior (Neumann, 2002; Ebner et al., 2005) and in fear processes (Viviani and Stoop, 2008) and early-life changes in the hypothalamus and in the amygdala may contribute to altered behavior and sensitivity to other challenges. Oxytocin functioning, especially in the amygdala has been related to autism (Bartz and Hollander, 2008; Meyer-Lindenberg, 2008) and an altered oxytocin function as a consequence of early-life experiences may therefore be of interest in the etiology of autism spectrum disorders. The results summarized herein show that MS may be utilized as an experimental model in further studies of the role of oxytocin in the early environmental impact on brain function, behavior, and pathology.

The advantage with animal experimental studies as those described in the present article is that the early environment can be manipulated and that the neurobiological consequences can be studied in detail under controlled conditions. The interactive effects of rearing conditions and later challenges can be studied. Of relevance for AUD is the possibility to study the effects of ethanol consumption in individuals with different histories of early-life experiences and distinguish between long-term effects caused by environmental factors in juveniles from the effects of ethanol consumption in adolescents. The interactive effects of early-life experiences and genotype can be studied using rodents with genetic ethanol preference such as described in studies of the ethanol-preferring AA rats (Roman et al., 2003, 2005). The epigenetic mechanisms underlying early-life impact on the brain can be delineated. Genes of interest for vulnerability or resilience to AUD can be identified in humans and the interactions with environmental factors can be further studied in genetically modified animals. Along with the development of more advanced gene technology we can insert/delete/modify genes of interest in rodents and examine the detailed mechanisms of environmentally induced alterations in gene activity in neurobiological and behavioral studies.

In conclusion, experimental studies have provided evidence that early-life events cause long-term alterations in neuropeptide circuits, especially oxytocin and the opioid peptide MEAP. The results indicate a link between early-life rearing conditions, opioid levels, and ethanol consumption and show that the ethanol-induced effects and the treatment with opioid antagonists later in life are dependent on early-life experiences. Endogenous opioids are therefore of interest to further study in the early-life impact on individual differences in vulnerability to AUD and treatment outcome. It is also evident from the studies presented herein that it is highly important to be particulate in the design and description of experimental conditions in MS studies. MS-induced effects on neuropeptides were dependent on the length of separations, whether the rat pups are kept together or placed individually during the separations, on the age when the effects are analysed and whether males or females were examined. Today there are a number of different protocols in use and provided that the experimental conditions are clearly described and motivated the plethora of protocols is not an obstacle but rather an advantage; they facilitate the choice of an appropriate experimental design depending on the question addressed.

### Conflict of interest statement

The authors declare that the research was conducted in the absence of any commercial or financial relationships that could be construed as a potential conflict of interest.
